# Animal Models of Subjective Tinnitus

**DOI:** 10.1155/2014/741452

**Published:** 2014-04-16

**Authors:** Wolfger von der Behrens

**Affiliations:** Institute of Neuroinformatics, University of Zurich and Swiss Federal Institute of Technology Zurich, Winterthurerstrasse 190, 8057 Zurich, Switzerland

## Abstract

Tinnitus is one of the major audiological diseases, affecting a significant portion of the ageing society. Despite its huge personal and presumed economic impact there are only limited therapeutic options available. The reason for this deficiency lies in the very nature of the disease as it is deeply connected to elementary plasticity of auditory processing in the central nervous system. Understanding these mechanisms is essential for developing a therapy that reverses the plastic changes underlying the pathogenesis of tinnitus. This requires experiments that address individual neurons and small networks, something usually not feasible in human patients. However, in animals such invasive experiments on the level of single neurons with high spatial and temporal resolution are possible. Therefore, animal models are a very critical element in the combined efforts for engineering new therapies. This review provides an overview over the most important features of animal models of tinnitus: which laboratory species are suitable, how to induce tinnitus, and how to characterize the perceived tinnitus by behavioral means. In particular, these aspects of tinnitus animal models are discussed in the light of transferability to the human patients.

## 1. Introduction

Subjective tinnitus is the phantom perception of sound in the absence of an external stimulus. In 1–3% of the general population it constitutes a significant impairment of the quality of life [1]. Despite significant research efforts, our understanding of the underlying neuronal mechanisms is far from complete. As a result the only approved therapies are symptomatic. One major obstacle arises from the fact that by its very nature tinnitus is a subjective phenomenon, and the only possible diagnosis relies on self-reports of the subjects [2]. This fact poses a problem not only for diagnosing tinnitus and identifying subtypes in human patients but also in animal models of tinnitus. At present, however, only research on animal models can provide us with the necessary understanding of the peripheral and central mechanisms that lead to the aberrant neuronal activity ultimately perceived as tinnitus. One proposed mechanism is that the pathological activity originates from plastic changes of the central auditory system following damages to the periphery. In a healthy system, this plasticity is essential for adjusting neuronal activity to changing acoustic environments. An acoustic trauma damaging the cochlea leads to a loss of input to the central stages of the auditory processing hierarchy. The lack of input is then overcompensated by increasing the spontaneous activity and neuronal synchrony. This proposed mechanism makes tinnitus a “plasticity disorder” [3] and it is this plasticity that should be targeted for treating tinnitus.

Results from animal models of tinnitus are an essential element in the combined efforts of different audiological specializations for developing new tinnitus therapies. The irreplaceable advantage of animal models lies in the possibility to study small neuronal networks and individual nerve cells through invasive methods such as extra- and intracellular recordings in potentially genetically engineered or sound exposed subjects. These means provide high spatial and temporal resolution (i.e., micrometer and millisecond range, resp. [4]) which is impossible in human studies applying electroencephalography or functional magnetic resonance imaging (exceptions are recordings during brain surgery). In fact, current hypotheses about the pathogenesis of tinnitus are mostly based on results from animal models, in particular from studies on tinnitus following noise-induced hearing loss [1]. However, since tinnitus is a conscious percept [5], many aspects have to be studied and characterized in laboratory animals through behavioral means. Furthermore, physiological measurements of tinnitus-related neuronal activity should ideally be sampled in awake animals in order to exclude artefacts from anesthesia and to facilitate a comparison with human subjects who only perceive tinnitus when awake. In summary, any behavioral assessment of tinnitus in the animal model should try to mimic as closely as possible conditions under which tinnitus develops in humans.

The first section of this review provides an overview of the different species used as animal models in tinnitus research. Then, the different methods used for tinnitus induction in animal models are reviewed. Finally, the competing behavioral paradigms used for assessing subjective tinnitus in the animal model are discussed. This sequence reflects a natural order of the main decisions to be made when designing animal experiments. Which species mimics the human condition and pathology best? What is the most appropriate way to induce tinnitus? Which behavioral paradigm is best suited for addressing the research questions?

## 2. Species Used for Behavioral Testing of Tinnitus

The first behavioral test for tinnitus in an animal model was established by Jastreboff et al. [6, 7] in 1988 using rats. Since then a number of different laboratory animal species and various strains have been used for the behavioral assessment of tinnitus. Besides the laboratory rat (*Rattus norvegicus*) [8–51], these include the domestic house mouse (*Mus musculus*) [52–58], the chinchilla (*Chinchilla laniger*) [59, 60], the Syrian golden hamster (*Mesocricetus auratus*) [61–64], the guinea pig (*Cavia porcellus*) [65–67], and the Mongolian gerbil (*Meriones unguiculatus*) [68, 69]. Since the early studies by Jastreboff et al., the laboratory rat remains the most prominent species used for investigating tinnitus at the behavioral level. However, an increasing number of studies are being performed on mice since the wide range of genetically modified strains is not available for rats at present. Comparing the hearing abilities and the suitability of different species for tinnitus assessment reveals advantages and drawbacks of the different approaches.

### 2.1. Hearing Ranges of Different Species

Compared to research on other sensory systems (e.g., the somatosensory modality which is usually investigated in the rat or mouse barrel cortex), investigation on hearing in mammals is characterized by a larger variety of established animal models. Usually, the criteria for selecting one species over another are not documented in the literature, even though this choice has serious consequences for the interpretation of results and their transferability to human subjects. Despite the fact that all species mentioned above belong to the same systematic order (*Rodentia*), their acoustical and behavioral ecology and physiology varies significantly from one another, and more importantly from the final subject of interest, the* Homo sapiens.* These differences are revealed most clearly by comparing the different audiograms. In rodents, the hearing range mostly covers the high frequency range beyond the upper human limit (highest audible frequency at 60 dB SPL for human is 17.6 kHz [70], rat: 58 to 70 kHz [71, 72], mouse: 85.5 kHz [70], chinchilla: 33 kHz [73], hamster: 46.5 kHz [74], guinea pig: 50 kHz [75], and gerbil: 58 kHz [76]). The same applies to the low frequency hearing limit (at 60 dB SPL in humans: 0.03 kHz [74], rat: 0.52 kHz [71], mouse: 2.3 kHz [70], chinchilla: 0.05 kHz [73], hamster: 0.096 kHz [74], guinea pig: 0.05 kHz [75], and gerbil: 0.032 kHz [74]). It has been proposed that mammals that do not hear below 0.5 kHz do not use temporal encoding for pitch perception [74], with the exact frequency of this boundary being discussed. This suggests that the two most widely used behavioral models of tinnitus, rat, and mouse employ neuronal mechanisms for pitch perception that fundamentally differ from those of humans. It has been argued that this difference applies only to the lower frequency range (<5 kHz). Nevertheless, interpretation of animal studies in relation to a human disease would be more directed in species with audiograms similar to humans (e.g., gerbil or chinchilla). However, gerbils are prone to a certain degenerative disorder of the auditory system, at least when supplied by a commercial manufacturer [77]. This caveat has to be taken into account when considering the gerbil as a potential model for subjective tinnitus.

Furthermore, choosing an animal model with human-like audiograms would facilitate the comparability of tinnitus pitch. Many studies cited above induced tinnitus through a noise trauma centered at 16 kHz. This treatment is presumed to give rise to a phantom percept that has a higher frequency than the region of highest sensitivity in the rat (around 8 kHz [71, 72]). In the mouse studies mentioned above the tinnitus inducing noise is centered at 16 kHz as well. In contrast to the rat, the mouse has its highest sensitivity at 16 kHz [78]. In humans, the average tinnitus pitch is in the range of 5–8 kHz [79] and the highest sensitivity lies around 3 to 4 kHz [80]. Independent of the species this means that the tinnitus-inducing stimuli have to be carefully matched to the hearing range of subjects in order to achieve comparability with the human pathology.

### 2.2. Differences between Rats and Mice in Suitability for Behavioral Paradigms

In recent years, the mouse has become a widely used behavioral model for tinnitus research. One of the reasons why mice entered the scene so late could be their presumed limited cooperation in behavioral training paradigms assumed from the larger variability in the effects found in acoustic startle experiments. Characteristically, all mouse behavioral studies of tinnitus mentioned above use a paradigm (gap-startle paradigm, introduced in 2006 by Turner et al. [8]) that does not necessarily require a functional auditory cortex [81] and does not require any behavioral training beyond adaptation to the setup [8]. However, so far no evidence has been published that substantiates the cognitive difference between rats and mice. On the contrary, in the somatosensory modality, rats and mice exhibit similar performance levels and learning curves when facing a complex 2-alternative forced choice task, which requires the discrimination of simultaneously presented whisker deflections at different frequencies [82]. The big advantage of the mouse model is the almost infinite range of genetically modified strains available. This allows the recording and manipulation of specific types of neurons (e.g., excitatory pyramidal neurons or inhibitory neurons of a certain cortical layer). However, so far no one has taken advantage of this feature of the mouse model. The downside, however, is that some mouse strains exhibit elevated auditory thresholds (measured as auditory brainstem responses) within 2 months after birth [83], a problem that may be aggravated in genetically modified lines. Rats do not exhibit this early onset of age-dependent hearing loss [84, 85].

## 3. Established Ways of Tinnitus Induction in Animal Research

Comparable to the diversity of species used in behavioral testing of tinnitus, there is a number of different ways of inducing tinnitus in animal models. In principle, there are two ways of inducing tinnitus. One way is through pharmacological means. Alternatively, tinnitus is induced by presenting high level stimuli for one hald to two hours. Both approaches try to mimic the etiology of tinnitus in humans even though the pathogenesis of subjective tinnitus remains poorly understood. However, it is commonly accepted that in many cases it commences with noise-induced damage to the hair cells of the inner ear, followed by deafferentation and hearing loss [86]. Such a trauma leads then to the initiation of compensatory processes in the central nervous system. In the healthy system, these processes warrant an activity level that is optimal for encoding the present acoustic environment. However, after a trauma and consequential deafferentation, this beneficial plasticity of the auditory system goes astray and overcompensates the missing input from the damaged region of the cochlea, leading to a permanently present phantom percept [87]. Hence, maladapted plasticity may underlie tinnitus and not the peripheral damage itself [88].

### 3.1. Pharmacologically Induced Tinnitus

The first study assessing subjective tinnitus in an animal model used a pharmacological method for induction [6]. The main advantages of a pharmacological tinnitus induction are its potential reversibility and its previous use in human subjects for inducing tinnitus as well (i.e., 3.9 g salicylate/day for 5 days [89, 90]). The two most commonly used substances where tinnitus is assessed by behavioral means in animal models are salicylate [9, 11–13, 16–20, 22, 32, 33, 35, 36, 38, 39, 43–45, 47, 50, 57, 58, 67] and quinine [10, 12, 18], an antimalarial drug. Other ototoxic drugs that have been investigated in animal studies are cisplatin (cis-diammine-dichloroplatinum (II)) and carboplatin [60]. Both are chemotherapeutics, with cisplatin predominantly targeting the outer hair cells [91] and carboplatin most likely affecting the inner hair cells [92]. Salicylate, the active component of Aspirin, is the most commonly used drug in animal models [93]. Therapeutically it is administered usually as a mild analgesic or in anti-inflammatory therapy (e.g., against rheumatic arthritis). Salicylate has the advantage of fast induction within minutes and its effects reverse within 72 hours of the last administration [94, 95]. In most studies cited above, salicylate was administered systemically, either orally or by injection. In some cases, salicylate was applied locally to the inner ear [39, 50] or central structures (e.g., auditory cortex) as well [34].

Salicylate most likely exerts its effects on hearing at high doses, both in the sensory periphery and in the central nervous system. In the auditory periphery it mainly targets outer hair cells, inhibiting their electromotility most likely by partitioning into the membrane [96] and blocking the prestin protein [97]. The consequence is a reduced cochlear sensitivity which manifests itself in a reduction of otoacoustic emissions (spontaneous and evoked), a decreased neural output, and ultimately a temporary hearing loss [94]. Long-term application of salicylate, however, leads to an increased expression of prestin, most likely as a compensatory reaction [98].

Parallel to these effects on the sensory epithelium, there is strong evidence that salicylate affects the central nervous system as well. Different levels of the auditory pathway have been identified as being modulated by salicylate. Amongst others these are the cochlear nucleus (CN), the inferior colliculus (IC), the medial geniculate body (MGB), and the auditory cortex (AC) [94]. The observed effects can either originate from changes of the input (i.e., altered cochlear output) or from direct action on the neuronal activity. In particular, it has been shown that different parts of the inhibitory GABAergic neurotransmission can be modulated by salicylate [94] and that a modulation of the GABAergic inhibition reduces salicylate-induced ototoxicity [50]. After chronic systemic administration, salicylate causes an increase in the expression of the GABA-synthesizing enzyme GAD [99]. In slice preparations, salicylate decreases GABAergic inhibition of auditory cortical pyramidal neurons, potentially facilitating hyperactivity [100]. These pieces of evidence indicate that acute salicylate administration reduces the GABAergic inhibition in the network, which is then compensated by an increased GABA synthesis. Other effects of salicylate are a reduced spontaneous firing rate in the inferior colliculus [101], adjustments in the tonotopy of the auditory cortex [102], and changes in the cochlear nucleus [103]. However, GABAergic transmission is most likely not the only target of salicylate. Another very likely target is the NMDA receptor (N-methyl-D-aspartate) [38, 104]. Finally, it has been proposed that salicylate acts on the extralemniscal pathway while noise trauma induces tinnitus in the lemniscal pathway [105].

The applied dosage of salicylate varies significantly between studies and species. However, it seems that with the right dosage (100 mg sodium salicylate/kg/day for two consecutive days) there is a reliable tinnitus induction, as shown with a behavioral test in rats [12]. How such a dosage in rats translates to a comparably critical serum level in humans is a source of uncertainty. In humans average salicylate serum concentrations of approximately 300 mg/L induce tinnitus [90]. 90 minutes after an i.p. injection of 350 mg/kg sodium salicylate (corresponding to 300 mg/kg salicylic acid), the salicylate serum level in the rat was 625 mg/L [106]. For the dosage of 100 mg/kg inducing reliable tinnitus in the rat, the expected salicylate serum concentration is approiamtely 56 mg/L. These differences (56 mg salicylate/L in rat vs 300 mg salicylate/L in human serum concentration) might indicate a higher sensitivity of the rat, differences in underlying clearance mechanisms, or different threshold criteria and administration schedules.

### 3.2. Tinnitus Induced by Acoustic Trauma

The second established method for inducing tinnitus in behavioral models is through acoustic trauma [8, 15, 20, 21, 23, 25–27, 30, 31, 37, 39–42, 46–49, 51–56, 59–66, 68, 69, 107]. It is assumed that a cochlear damage is in most cases the trigger for a sequence of events leading to the development of tinnitus in humans. However, not every hearing loss resulting from a trauma gives rise to tinnitus and a subset of patients exhibit normal audiogram indicating that “hidden hearing losses” play a role as well [108]. Acoustic trauma and subsequent hearing loss induces a number of acute and chronic changes in the periphery and the central nervous system. At the periphery, an acoustic trauma results in outer hair cell damage, cochlear dead regions (no functional inner hair cells) [109], damaged stereocilia in both types of hair cells [110], and deafferentation of auditory nerve fibers [111]. Typically, the hearing loss accompanying tinnitus is located in the high-frequency range. The tinnitus pitch itself is either near the edge of the hearing loss or in the frequency range of the damaged region itself [112].

The parameters for inducing tinnitus through acoustic trauma in the animal model are quite variable. Typically, a high level noise stimulus is applied for 1 to 2 hours under anesthesia, either to one or both ears. For the rats, a widely used stimulation paradigm consists of an octave-band noise with a peak intensity of 116 dB sound-pressure level centered at 16 kHz for 1 hour [8]. However, sound level (80 dB SPL [62] to 130 dB SPL [39], [63]), duration (2 min [25] to 7 hours [28]), frequency (2 kHz [69] to 22 kHz [52]), frequency range (pure tones [27] to broadband noise [15]), and concerned ear (uni- or bilateral) vary a lot between studies. In rats, binaural exposure to a 10 kHz tone for 1-2 h leads to significant tinnitus when the sound level was 120 dB but not at 80, 100, or 110 dB SPL [51].

The primary criteria for selecting the stimulus parameters are usually the hearing range of the species, the targeted tinnitus pitch, and time course (temporary versus chronic). Mice exposed to noise centered at 16 kHz at 116 dB SPL for 1 hour exhibited signs of tinnitus for 25 months afterwards [54], while in rats exposed to 17 kHz pure tones at 115 dB SPL for 2 minutes tinnitus lasted only 13 min [25] (induction under isoflurane anesthesia). In gerbils, a reliable and chronically induced tinnitus can be achieved by noise stimuli with an exposure time of at least 1 hour and narrow bandwidth leading to a temporary threshold shift and ultimately to self-sustaining activity perceived as phantom sound. Such a protocol leads to a hearing loss that disappears after 3 to 6 weeks and a tinnitus percept centered at the center-trauma frequency appearing 5 to 7 weeks after induction [68]. Hamsters exposed to a 10 kHz tone at 110 dB SPL for 4 h exhibited tinnitus symptoms within one day after exposure [62] indicating the possibility of an almost immediately tinnitus onset after acoustic trauma.

The changes after acoustic trauma at the different stages of the ascending auditory pathway are manifold and complex. Within hours after an acoustic trauma, the spontaneous neuronal activity in the primary auditory cortex (A1) of the cat increases in the frequency region below the damage [113]. This increase presumably originates from a loss of inhibition from the cortical regions representing frequencies of the cochlear damage. Weeks after an acoustic trauma, the tonotopic map of A1 reorganizes so that there are no neurons with characteristic frequencies above the frequency of the traumatizing stimulus [114]. In parallel, the activity in the auditory cortex becomes more synchronous after acoustic trauma [113]. Neurons in the inferior colliculus exhibit increased spontaneous firing rates after an acoustic trauma [60]. In the dorsal cochlear nucleus (DCN) an acoustic trauma induces an increase in spontaneous activity which correlated with the strength of the behavioral tinnitus evidence [63] and specifically in fusiform cells [59]. However, DCN ablation does not change the psychophysical indicators of tinnitus [21].

### 3.3. The Role of Anesthesia

While salicylate can be administered for tinnitus induction in awake animals, it is usually anesthetized for tinnitus induction through acoustic trauma. The anesthesia is either injectable (very often a combination of ketamine and xylazine, or pentobarbital) or an inhalable one (usually isoflurane). How different anesthetics influence the development of hearing loss and tinnitus after acoustic trauma is largely unknown. However, isoflurane has been shown to diminish the amplitude and duration of temporary tinnitus after a short exposure to loud sounds [25]. Under pentobarbital, isoflurane, or halothane anesthesia noise-induced hearing loss in mice is less (62.5 dB, 45.5 dB, 39.3 dB threshold increase, respectively) compared to the unanesthetized control group (77.5 dB threshold increase) [115]. In addition, the influence of anesthesia on any electrophysiological recordings has to be taken into account, as anesthesia influences the receptive fields and the spontaneous activity of the rat auditory cortex [116].

### 3.4. Summary Tinnitus Induction

The advantages of a pharmacological induction of tinnitus with salicylate are the following. Salicylate has a fast onset and is metabolized within hours to days. It can be tested in human subjects as well as in animal models. Salicylate administration can be locally confined either to the cochlea [50, 117] or to specific brain structures and systemic administration is possible without anesthesia. The drawbacks are a presumed multitude of mechanisms giving rise to tinnitus, a lackof specificity interms of the locus of action, tinnitus pitch (0.9 to 14.5 kHz [118]), and relevance for the human pathology since in humans it is usually triggered by noise trauma. Furthermore, salicylate does not induce chronic tinnitus as it recedes when the intake is stopped. These aspects hinder the identification of neuronal substrates involved in the pathogenesis and maintenance of human tinnitus by means of salicylate.

One advantage of inducing tinnitus through acoustic trauma is the possibility to induce unilateral tinnitus, allowing the animal to serve potentially as its own control as done in some studies (e.g., Turner et al. [55]). However, one has to keep in mind that the ascending auditory pathway is characterized by significant binaural projections on every stage. Even if the tinnitus is perceived unilaterally, it is manifest in contra- and ipsilateral instances. Therefore, real controls (i.e., animals not exposed to noise as done by Turner et al. [55]) are required as well. Another advantage of tinnitus induction by acoustic trauma is the fact that this is most likely the most common form observed in human patients [1]. One of the biggest uncertainties when inducing tinnitus through an acoustic trauma is the resulting percentage of animals exhibiting tinnitus in behavioral tests. These numbers vary significantly in the literature, according to Knipper et al. [119] from 30% to 80%.

Ultimately, the choice of how to induce tinnitus in a behavioral study depends on the research question and which form of tinnitus will be studied. It has to be kept in mind that an acoustic trauma and drugs induce tinnitus through different mechanisms [120] and that both methods have certain methodological constrains (e.g., that an acoustic trauma very often has to be applied under anesthesia, depending on local animal welfare regulations).

## 4. Behavioral Models for Assessing Tinnitus in Animals

Diagnosis of subjective tinnitus in human patients relies almost exclusively on the self-report as there is no external sound source present and it manifests itself only in the neuronal activity of subject's brain. There are some noninvasive approaches that provide potentially objective measures for subjective tinnitus by means of functional magnetic resonance tomography, electroencephalography, magnetoencephalography, and positron emission tomography [121]. However, at present none of these methods is applied routinely for diagnosing tinnitus and it is unknown whether the observed effects are directly caused by tinnitus or by the emotional stress usually accompanying severe tinnitus. This challenge of diagnosing tinnitus poses a supreme obstacle for developing an animal model with behavioral evidence of tinnitus. Nevertheless, a reliable behavioral assessment of tinnitus in the animal model is essential for understanding the pathology and the development of therapies. In typical behavioral tests performed in sensory physiology, the presence of a stimulus has to be detected or stimuli have to be discriminated and the animal's decision is indicated by a nose poke or a lever press. The absence of a stimulus usually requires no specific response, as seen in go/nogo paradigms [122]. A continuous phantom percept like tinnitus hardly fits into such a framework of psychophysical experiments, as it is assumed to abolish the notion of silence [105]. Since the first publications by Jastreboff et al. [6, 7] 25 years ago, a number of different behavioral paradigms for addressing this issue have been developed. Any behavioral assessment of tinnitus has to consider the confounding influences of possible hearing loss (after noise trauma) and hyperacusis accompanying tinnitus induction. Furthermore, an ideal test for tinnitus in animals would be closely modeled on tinnitus tests performed in humans and might even be applicable to humans as well.

### 4.1. Conditioned Avoidance Paradigms

Jastreboff et al. [6, 7] used a standard learning technique, the Pavlovian conditioned response suppression by the induction of fear [123]. Water-restricted animals were exposed to a constant background noise (approximately 40 dB SPL) during which they were allowed to collect water from a drinking tube. The conditioned stimulus (CS) was the offset of the background noise for 30 s. The behavioral readout was the ratio of licks during the CS compared to the number of licks in the period preceding the silent gap (suppression ratio). During suppression training the CS periods were terminated with an inevitable foot shock as unconditioned stimulus (US). This led to the extinction of licking during the CS. The training was continued until the suppression ratio was below 0.2. Next, animals were injected with salicylate in order to induce a phantom sound that was assumed to fill out the silent gap of the CS. During the testing there was no foot shock (US) and the response suppression extinguished over time. In salicylate-treated animals the response suppression extinguished within 2 days, while it took saline-injected animals 4 days until the response suppression was extinguished. The faster extinction time course in salicylate-treated animals has been interpreted as an indicator of tinnitus as the animals did not perceive the silent gaps (CS) anymore. The most important control of this study was a group of animals that received salicylate before the suppression training. These animals associated the tinnitus perceived during the silent gaps with the foot shock. Consequently, during the testing sessions, when no foot shock was given, the animals stopped licking during the silent gaps as they associated the tinnitus with punishment and the extinction took longer. This rules out the possibility that salicylate by itself changed the behavior in some ways (e.g., increased thirst, altered impulsivity). Hearing loss after salicylate administration as an explanation for the faster extinction was ruled out since reducing the amplitude of the continuous noise by 20 dB did not lead to a faster extinction.

Heffner and Harrington [61] modified this conditioned response procedure and tested hamsters for tinnitus. They aimed at a protocol that allows to measure behavioral indicators of tinnitus in individual animals. The basic paradigm again consisted of a broadband noise during which the animals were allowed to drink (safe signal) and silence during which the animals had to stop drinking. In training, the animal was shocked if it contacted a water spout during a silent period. Animals were trained for 32–35 sessions in order to achieve a performance above 70%. Performance was calculated as the average percentage of time the animal contacted the spout during noise and was not in contact during silence. The tinnitus was induced by a pure tone acoustic trauma (10 kHz, 124 dB SPL for 4 h) applied to the left ear. During test sessions (5 days after acoustic trauma), there was no shock when the animal contacted the spout during silent periods. As in Jastreboff et al. [6, 7] the time course of the extinction of the response suppression during silent periods was indicative of the perceived phantom sound. Animals receiving a pure tone trauma were more likely to drink during silent periods compared to a control group. This difference was visible in performance scores of individual animals as well. However, the variability was quite big and there was a certain overlap in the distributions of performance scores of the control group and the one that received a trauma.

Similar conditioned suppression paradigms have been used in other studies as well (e.g., Zheng et al. [44]). The main advantage of their approach is that it can be applied easily to larger numbers of animals since the training period is quite short. Different tinnitus induction protocols have been proven to be effective with such paradigms which allow pitch and amplitude of the tinnitus to be characterized. Its major drawback is a relatively short period for actually assessing the tinnitus. Since the indication for tinnitus is the time course of suppression extinction (no foot shock), only short time spans (days) can be monitored and a more detailed analysis of the tinnitus over time is impossible.

Bauer and Brozoski [37] published an operant conditioning approach for measuring tinnitus in the animal model (rats). Here, subjects were trained to lever-press in order to receive a food reward when an auditory test stimulus was present (60 dB SPL broadband noise or pure tones). During silent periods, the animals had to stop lever pressing. A running index of lever press behavior was computed for windows of 1 min length. If the animals kept lever pressing in the silent periods, they were punished with a foot shock if they met or exceeded a certain criterion of the running index. In the testing sessions, pure tones of different frequencies and amplitudes were presented as well as silent gaps. Lever pressing during pure tone presentation was not punished; however, pressing during silent gaps was still punished. The discrimination functions (pure tones and silence) of animals receiving an acoustic trauma (noise centered at 16 kHz, 1 octave bandwidth) and unexposed control animals (or animals with a simulated hearing loss through ear plugs) differed significantly. This has been interpreted as an indicator for tinnitus as the traumatized animals could not differentiate between test tones and real silent gaps which were “filled” with the phantom sound. Since the behavioral contingencies were the same during testing and training, it was possible to measure the tinnitus induced by noise trauma over extended periods (up to 17 months). Additionally, the tinnitus properties (pitch, loudness) could be measured in detail, as Bauer and Brozoski [37] identified the tinnitus pitch at 20 kHz. The downside of this approach is that it requires careful training and can take extended periods of time for the animals to reach criterion before the actual testing takes place.

A slightly different approach was published by Lobarinas et al. [11]. Rats were put on a food restriction schedule and received a food pellet in regular intervals. This scheduled food intake induced polydipsia leading to a constant licking for water between the food deliveries. Sound stimuli were paired with a foot shock and silence periods were the “safe signal” for drinking. The behavioral readout is the number of licks during silent periods. Animals perceiving a phantom sound are expected to lick less during quiet periods as they try to avoid a foot shock. The motivation to develop such a schedule-induced polydipsia avoidance conditioning paradigm was to assess tinnitus in individual animals and over extended periods of time. IIn order to achieve a performance of >90% of licks during quite periods the animals were trained for 2-3 weeks. Another study confirmed the sensitivity of this test for tinnitus by measuring it with different paradigms as well [19]. Lobarinas et al. [11] were able to monitor salicylate-induced tinnitus and recovery over 40 sessions.

### 4.2. Positive Reinforcement Paradigms

An operant paradigm with positive reinforcement has been proposed by Rüttiger et al. [16] which reduced the need for punishment through foot shocks to a minimum. Again, a continuous noise was a safe signal for the rat to access one of two water spouts in order to receive a reward (3% sucrose in water). The rat had to switch from one spout to the other in order to collect a reward. If the animal accessed one spout during a silent period, no reward was delivered and a foot shock is applied. During testing for tinnitus, there was no reward and no punishment during the silent gaps. In order to still get useful behavioral responses, even before testing for tinnitus, only a percentage of switches between reward spouts were rewarded. This prolonged the time to extinction of the discriminative behavior between noise and silent gaps. It should be emphasized that the foot shock in this study was quite weak and avoidable and the behavior of the animals was most likely driven by the reward value of the sugar water itself. The reinforced behavior was activity (alternating between spouts). Tinnitus was induced with an injection of salicylate (350 mg/kg bodyweight) after the animals achieved a certain performance level (12 to 15 sessions before administration). Testing took place immediately after tinnitus induction in order to characterize the immediate effects of salicylate. The behavioral indicator was the ratio between number of reward spout access during noise and during silence, divided by the ratio between noise duration and silence duration. After salicylate treatment, the number of access to the reward spouts during silent periods increased relative to the access during noise presentation. This paradigm has been used in a couple of follow-up studies, where the tinnitus was induced through an acoustic trauma, emphasizing its robustness and applicability to a wider range of tinnitus models [42, 51, 119].

Another paradigm using only mild electric shocks and positive reinforcement was published by Heffner and Koay [62]. Here, hamsters received a unilateral acoustic trauma and were trained to localize a sound source (left or right) in order to collect a reward at that side. Responses to the wrong side were shocked. During training, sound trials were interleaved with a few silent trials (catch trials) which were not punished or rewarded. These trials served as an indicator for the animal's side preference. After the acoustic trauma, the side preference shifted to the side where the trauma was applied. This was interpreted as a result of a phantom sound perceived by the animals, as they were trained to go to the side where a stimulus was localized. In summary, the operant conditioning paradigms described here usually require a very careful and time-consuming training of the animals. However, this is compensated by the possibility to test animals repeatedly and over extended periods.

One very recently published paradigm does not apply any aversive stimulus at all but only positive reinforcement through food pellets [47]. Here, the rats had to press one lever in the presence of a sound (tone lever) and press another lever in the absence of sound (0 Hz lever). After treatment with salicylate (75, 150, 300, or 450 mg/kg body weight) or exposure to intense sounds (140 dB SPL at 4 kHz for 4 hours) the animals exhibited an increased number of “tone lever” presses in the absence of any sound. This increase was ascribed to the presence of the tinnitus phantom sound. Again, the extensive training required (2-3 months) by this paradigm is balanced by the possibility to test animals over extended periods.

A navigation approach was pursued by Guitton and Dudai [39]. Here, the rats had to swim in a water T-maze and find a hidden platform. The platform was in one of the two arms of the maze if a tone was presented and in the other arm, when no tone was presented. Two measures were taken for quantifying the sound perception of the animal: time spend in one arm of the maze and percentage of correct choices. After 3 days of training the animals reached the correct arm in 80% of the cases within an average time of 4 s. After an acoustic trauma approximately half of the rats (12 out of 26) behaved as if they perceived in tone even when there was no sound present (measured as an increased time spent in the arm associated with the tone).

### 4.3. Gap Startle Reflex Paradigm

During the last years, a completely different and objective paradigm was established for measuring tinnitus in laboratory animals. It is based on the acoustic startle reflex or response (ASR) which is a very rapid contraction of skeletal muscles following the presentation of acoustic stimulus with high intensity [124]. The central pathway for this startle response is well described and involves only three synapses. The cochlear input is relayed through the brainstem to the pedunculopontine tegmental nucleus and the nucleus reticularis pontis caudalis which initiates the startle response [105]. The amplitude of this response is modulated by many factors like fear potentiation and sensitization. In particular it can be reduced by a preceding stimulus or silent gap in a continuous background noise. The basic idea for tinnitus detection is that a phantom sound can mask these gaps. In animals experiencing tinnitus, the acoustic startle reflex is not diminished even when preceded by a gap. This concept was first tested and published by Turner et al. [8] as a new approach to efficiently test for tinnitus in the animal model. To this end rats received an acoustic trauma (unilateral 16 kHz octave-band noise at 116 dB SPL, under anesthesia). Next, animals were placed in a testing chamber where a continuous background noise was presented (centered at 10 or 16 kHz or broadband noise, 60 dB SPL). The animal's response was measured as force applied to a Piezo transducer in the floor of the chamber. The startle stimulus was a 115 dB SPL noise burst for 20 ms. Half of the startle stimuli were preceded by a 50 ms gap in background noise which would reduce the startle amplitude in naïve animals. Animals receiving an acoustic trauma exhibit less inhibition of the startle response when it was preceded by a gap compared to controls. However, this was only the case when the background noise was centered at 10 kHz and not at 16 kHz or for broad band noise. This result confirmed the previously characterized tinnitus pitch at 10 kHz which was determined by an operant conditioning paradigm [8]. Hearing loss was ruled out as possible explanation for this effect as a simulated unilateral hearing loss (ear plugs) did not change the inhibition of the startle response by a preceding gap.

This paradigm or some derivatives (e.g., measuring the Preyer reflex in guinea pigs by Berger et al. [67]) were adopted by many research groups [11, 20, 23, 46, 48, 49, 52, 53, 64, 65, 68, 69] because they offer a number of advantages. The main benefit for experimentalists is that it is a fast method in terms of training and testing. No training beyond test chamber adaptation is required and testing can take place in less than one hour, allowing high-throughput screening which is not possible with more complex conditioned behavioral paradigms. Additionally, the animals do not have to be on a restricted food or water schedule and the neuronal circuitry giving rise to the startle response is well described. Finally, this is a fairly objective measurement as the reflex is only to a certain degree modulated by top-down processes [125]. However, a number of issues have to be taken into account when considering a gap startle paradigm for assessing tinnitus in animal models. First, it is unknown whether in human tinnitus patients gaps are “filled” with the phantom percept. In the light of transferability of results from the animal model to humans, this is a major drawback and has been only very recently addressed by Fournier and Hébert [126]. This study explicitly tested gap inhibition of a startle response (eye blink) in tinnitus patients (high-pitched) in order to compare it to animal studies. The key finding was that tinnitus patients exhibited a similar change of startle response amplitude when preceded by a gap as the traumatized animals did in the studies mentioned above. Despite some differences in the results compared to the study by Turner et al. [8] (e.g., gap deficits occurred at high- and low-frequency background noise in humans but not in the animal study) this is evidence that the gap startle paradigm could be a valid model for studying tinnitus and that it measures manifestations of a phantom sound comparable to the one observed humans.

One objection put forward regarding the gap startle paradigm is its reflex nature and that it does not necessarily involve the auditory cortex. It has been shown that ablation of auditory cortex in mice does not change the gap startle response after one month compared to a control group. However, one day after cortex ablation there were differences, indicating a temporary modulatory effect of auditory cortex on activity in the brain stem circuitry that gives rise to the startle response [81]. Other studies in rats [127, 128] lesioning or deactivating the auditory cortex found changes for certain gap durations. Thus, the role of auditory cortex in the gap startle paradigm still remains to be elucidated. It has been hypothesized that the neural substrate of tinnitus involves an increase in spontaneous activity, an increase in neuronal synchrony, and a reorganization of the tonotopic map in auditory cortex [105, 120]. Testing this hypothesis ideally requires a behavioral paradigm, which necessarily involves the auditory cortex and not only a brain stem circuit. It has been shown that tinnitus patients and healthy subjects can detect gaps typically used in gap startle paradigms with similar performance [129]. This result indicates that changes in gap startle paradigms do not automatically mean that higher processing of these stimuli is impaired in tinnitus patients. Lobarinas et al. [49] put forward the potential influence of hearing loss on the gap startle response and tackle this concern twofold in a dedicated study: first, by optimizing the startle stimulus so that it was outside the range of the hearing loss and second, by substituting the broad band noise startle stimulus with a rapid air puff to the animal's back which cannot be subject to hearing loss. In particular, the air puff approach preserved the startle response, even after conductive hearing loss. However, its operational reliability for measuring tinnitus remains to be proven.

## 5. Summary

The ultimate benchmark for any animal model measuring subjective tinnitus is comparability to the human patient. Any researcher starting to model tinnitus in laboratory animals has to make a decision regarding the species, the method of tinnitus induction, and the behavioral test. The current review provides an overview over the most commonly used methods and approaches.

The most important criteria for choosing a certain species is its hearing range, its aptitude for behavioral studies and the availability of genetically modified strains. These strains allow the recording and manipulation of specific types of neurons revealing their role in tinnitus. The behavioral differences between the commonly used species are a source of uncertainty. The majority of studies discussed here were done in rats, considered to be well suited for behavioral testing even with more difficult sensory decision making paradigms [130]. Another advantage of the rat as an experimental model for studying the neuronal circuitry underlying tinnitus is the possibility to implant electrode arrays with high channel counts and perform chronic recordings in awake [131] and behaving animals (e.g., Otazu and Zador [132]). The disadvantage of the rat as a model is its high-frequency hearing range, which differs significantly from the human one. Still, it remains unclear so far if these differences in hearing rage are significant for the pathogenesis, perception, and potential therapy of tinnitus. Additionally, there are only a limited number of genetically modified rat strains available. However, this last factor is certainly changing in the future as more and more recombinase-driver rat lines are developed (e.g., [133]) and the establishment of the potentially universally applicable CRISPR genome-editing technique [134], which has already been applied successfully in cynomolgus monkey (*Macaca fascicularis*) [135].

The tinnitus induction protocol should model the human pathogenesis. For the majority of human cases, an acoustic trauma-induced hearing loss is suspected. This favors a tinnitus induction through acoustic trauma over a pharmacological induction. On the other hand, an induction through salicylate has the advantage of fast onset of tinnitus and its reversibility. This allows a behavioral setting that can be controlled for tinnitus related behavioral peculiarities of individual animals. Furthermore, salicylate can be applied locally which allows to study tinnitus-related changes at different stages of the auditory processing hierarchy. Whichever method is used, the accompanying hearing loss and hyperacusis have to be taken into account for interpreting the results. However, to disentangle tinnitus and hyperacusis is very challenging as they are comorbid. Very recently, it has been demonstrated that mice exposed to “neuropathic” noise displayed a hyperresponsivity to acoustic startle stimuli. At the same time the gap detection deficits (measured as prepulse inhibition of the startle response) were limited to certain gap-stimulus latencies which cannot be explained by the presence of a phantom sound which should fill the gap for all latencies [107] and which therefore has be interpreted as a potential indicator of hyperacusis.

The behavioral approaches testing for subjective tinnitus presented here include paradigms using reflexes, Pavlovian conditioning, and operant conditioning. Tinnitus in humans is a conscious percept which involves the auditory cortex [120]. It is usually measured through sensory decision making tests which can be applied over extended periods. A behavioral test for laboratory animals should be shaped along these aspects, in particular the cortical involvement and extended testing period. Additionally, such a test should only require limited training periods in order to achieve a high throughput. For conditioned responses the auditory cortex is not essential, as a cortical ablation does not prevent an animal from a classical conditioning response to simple tones [105]. However, more complex tones (e.g., frequency modulated tones) necessarily require a functional auditory cortex for discrimination [136]. More complex operant conditioning tasks most likely rely on an intact auditory cortex [105]. This has to be balanced with the usually more time consuming training protocols required for operant conditioning paradigms. For the conditioning paradigms introduced here, an involvement of the auditory cortex has not been shown yet, leaving an explanatory gap between the observed behavior and its neuronal substrate. Furthermore, modulation of the tinnitus percept through higher cognitive functions as demonstrated in humans (e.g., attention [137]) has been ignored in animal studies so far, most likely due to a lack of behavioral paradigms allowing the manipulation of these functions. However, a comprehensive animal model should ideally take this factor into account as well.
